# Weight Regain After Liraglutide, Semaglutide or Tirzepatide Interruption: A Narrative Review of Randomized Studies

**DOI:** 10.3390/jcm14113791

**Published:** 2025-05-28

**Authors:** Massimo Quarenghi, Silvia Capelli, Giulia Galligani, Arianna Giana, Giorgia Preatoni, Rosamaria Turri Quarenghi

**Affiliations:** 1 Clinical Nutrition and Dietetics, Department of Internal Medicine, Ospedale San Giovanni, Ente Ospedaliero Cantonale (EOC), 6500 Bellinzona, Switzerland; arianna.giana@eoc.ch (A.G.); giorgia.preatoni@eoc.ch (G.P.); 2 Clinical Nutrition and Dietetics, Department of Internal Medicine, Ospedale La Carità, Ente Ospedaliero Cantonale (EOC), 6600 Locarno, Switzerland; silvia.capelli@eoc.ch (S.C.); giulia.galligani@eoc.ch (G.G.); 3 Privat Medical Office, 6600 Locarno, Switzerland; rosyturri@hotmail.com

**Keywords:** glucacon like peptide (GLP1 RA), GIP/GLP-1 RA, tirzepatide, liraglutide, semaglutide, weight regain, bariatric surgery

## Abstract

**Objectives:** The primary objective of this review is to analyze the effects on body weight of discontinuing therapy with glucagon-like peptide-1 receptor agonists (GLP-1 RAs) or tirzepatide in patients treated for obesity. In recent months, there has been a considerable increase in the utilization of GLP-1 RAs and GIP/GLP-1 RAs. However, the paucity of available data regarding their medium- to long-term safety remains a salient concern. Of particular significance is the observation of the weight curve following their suspension, a subject that has received scant attention to date. **Methods**: For this, a bibliographic search was carried out in three electronic databases: PubMed, Cochrane Library and Google Scholar. The following filters were applied: **in the last 10 years, Randomized Controlled Trial, Adult: 19+ years. The review was restricted to randomized controlled trials to reduce bias and ensure the high quality of the studies examined.** A total of 427 references were identified, 178 articles were read in full, and 13 articles were included in the analysis. **Results and Conclusions**: The analysis showed a rapid regain of weight after cessation of therapy, regardless of the duration of the treatment with GLP-1 RA or GIP/GLP-1 RA. This rebound is likely to substantially mitigate the metabolic benefits attained through weight loss. Given the efficacy of these drugs, it is essential for future research to focus on elucidating the optimal duration of these treatments or identifying techniques or schemes that involve a reduction in dosages to prevent weight regain.

## 1. Introduction

The prevalence of overweight and obesity has been increasing on a global scale, substantially influencing both the health status of the population and the financial costs associated with healthcare [1,2,3]. It is estimated that an individual with obesity has a life expectancy that is several years shorter than that of a person with a healthy weight [4]. The treatment of overweight and obese individuals not only exerts an economic impact but also enhances quality of life and reduces morbidity and mortality [5,6].

Although the modification of lifestyle, specifically the improvement in the diet and the introduction of physical activity, still constitutes the cornerstone of obesity management, its ineffectiveness when implemented in isolation has been well documented [7,8]. In contrast, the efficacy of bariatric surgical therapy over a period of up to two decades post-surgery has been substantiated through data [4]. Bariatric surgery, now termed metabolic surgery, has been demonstrated to be cost-effective as well as efficacious in the treatment of obesity and in the management of certain co-morbidities, including the possibility of inducing remission of type 2 diabetes [9]. Nevertheless, it is not readily accepted by obese subjects, as it is an invasive procedure [10]. The conservative alternative treatment for obesity, involving only dietary modification and/or physical activity, as previously outlined, exerts a negligible impact on weight reduction, and this approach falls short of achieving the targets deemed necessary to impact morbidity and mortality [11].

Until a few years ago, the drug armory for treating obesity was limited both by the number of molecules available and by their modest efficacy [12]. Some drug molecules, such as orlistat and lorcaserin, have shown efficacy, albeit limited, in weight loss, but the data show that they do not prevent weight gain after cessation [13,14]. The identification of novel molecules for the treatment of obesity is therefore paramount in combating what is considered by the WHO to be a global epidemic [1,2].

In recent years, glucagon-like peptide-1 receptor agonists (GLP-1 RAs, e.g., semaglutide) and hybrid gastric inhibitory polypeptide (GIP) analogs and GLP-1 RAs (GIP/GLP-1 RAs, e.g., tirzepatide) have emerged as novel pharmacological agents for the treatment of obesity [15,16]. The introduction of GLP-1 RAs and GIP/GLP-1 RAs was met with immediate enthusiasm, heralding a transformative era in the treatment of obesity. This positive reception can be attributed to several key factors, including these drug’s ease of administration, effectiveness across diverse populations and ethnic groups, and capacity to enhance quality of life (health-related quality of life) through weight reduction with a short-term safety profile that is both acceptable and well established [17,18,19,20,21].

A substantial amount of data is being collected, demonstrating that the efficacy of these treatments extends well beyond their first year of use [17]. This progress enables a clearer understanding of the effectiveness of the therapy in promoting weight reduction over time, with particular emphasis on the initial weeks of utilization. As demonstrated in phase 2 and 3 studies, the weight reduction curve appears to be similar across the various molecules, exhibiting significant weight loss, particularly during the initial weeks and months. However, a “ceiling” is observed after around one year of use for GLP-1 RAs [22,23,24].

Direct comparisons of the relative effectiveness of molecules already on the market are also being initiated; however, the comparative value of the dosages employed in these studies is not always reliable: in some cases, the dosage of one molecule is often not directly comparable to that of other molecules [25,26]. The dosage of the molecule is of particular significance in evaluating its effectiveness, as evidenced by the findings of phase 2 and 3 studies, which have demonstrated that the efficacy of the various molecules in promoting weight loss is predominantly influenced by the dosage administered [27,28]. Consequently, a decline in the utilization of substandard and less user-friendly molecules, such as liraglutide, which have been available for a considerable time, is being observed in specific regions worldwide. In Switzerland, for instance, liraglutide is no longer covered by the healthcare system, resulting in the loss of valuable long-term safety data.

In recent months, there has been a considerable increase in the utilization of GLP-1 RAs and GIP/GLP-1 RAs, as evidenced by a substantial increase in the number of publications addressing their application. However, the paucity of available data regarding their medium- to long-term safety remains a salient concern. Of particular significance is the observation of the weight curve following their suspension, a subject that has received scant attention to date [17,29].

The focus of the present work is on weight regain upon stopping the therapy, as already reported in some studies [30]. A significant proportion of existing guidelines have not yet been updated to establish whether such therapies should therefore be maintained “ad vitam” [16]. The duration of drug treatment for obesity with molecules such as GLP-1 RAs or hybrids is not yet established, and there is a paucity of data on long-term safety. There are countries, such as Switzerland, where basic health insurance covers the costs of GLP-1 RAs for a maximum of 3 years [31]. This is a fundamental issue, given the high costs of these treatments [32].

Consequently, the present study was conceived with the objective of addressing this issue through a rigorous research approach. This was achieved by conducting a comprehensive analysis of the consequences of the suspension of therapies containing these drugs in randomized studies, in which patients were meticulously monitored after the cessation of therapy, followed by a comparison with the placebo.

## 2. Materials and Methods

### 2.1. Narrative Review Construction

The present narrative review was organized through the “Narrative Review Checklist”, which is provided by the Academy of Nutrition and Dietetics [33,34]. Thus, we carried out specific checks of the manuscripts’ structures and engaged in a careful selection of articles following the criteria described in the next section.

### 2.2. Study Selection

Two researchers performed a literature search on 6 December 2024, in three electronic databases: PubMed, Cochrane Library and Google Scholar. The pre-defined search terms were as follows: ((((((liraglutide) OR (semaglutide)) OR (tirzepatide)) OR (GLP-1)) AND (weight loss)) OR (weight regain)) NOT (bariatric[Title/Abstract]). The following filters were applied: in the last 10 years, Randomized Controlled Trial, Adult: 19+ years. The review was restricted to randomized controlled trials to reduce bias and ensure the high quality of the studies examined. Animal and experimental model studies were excluded from the review, and double entries were removed. A total of 427 references were identified by the literature search.

In accordance with the Preferred Reporting Items for Systematic Reviews and Meta-analyses (PRISMA) structure, two colleagues independently reviewed a total of 249 articles, initially determining their inclusion based on the title and abstract [35]. Following this preliminary evaluation, 249 articles were excluded from further consideration due to their failure to meet the predetermined inclusion criteria. Then, 178 articles were read in full, with 13 articles included in the analysis.

## 3. Results

**Figure 1:**
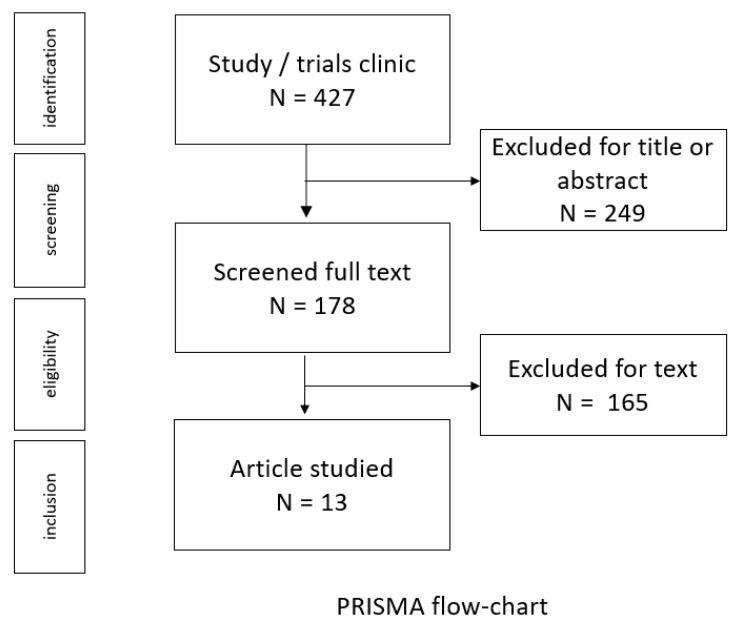
Flow diagram illustrating the process of our review.

A comprehensive literature search yielded 427 articles; however, following rigorous application of the inclusion criteria, as shown in the PRISMA flow chart, only 13 articles were deemed suitable for the analysis (Figure 1 and Table 1, Table 2 and Table 3) [35].

Of these, only five had a clear focus on an analysis of weight recovery against the placebo after stopping the drug therapy [Table 1], refs. [29,30,36,37,38].

The duration of the therapeutic intervention in the included trials ranged from several weeks to 160 weeks, as documented in the SCALE study [29]. It is important to note that four of the studies, two pairs of studies, are highly intertwined, using the same database and study population: the SCALE and STEP studies [29,30,36,37]. Consequently, it can be concluded that there are currently only three studies of a sufficiently high scientific standard to enable the research question to be addressed.

Over the course of the 20-week run-in phase of the STEP-4 study, in which patients without diabetes were administered semaglutide, a mean body weight reduction of 10.6% was observed [30]. From week 20 to week 68, when patients were randomized to receive a placebo or to continue semaglutide, a mean weight change of −7.9% was recorded in the semaglutide group, while a mean increase of 6.9% was observed in the placebo group (difference: −14.8 [95% CI, −16.0 to −13.5] percentage points; *p*  <  0.001). No data were collected for body weight at or following week 75. The STEP 1 extension study complemented STEP 4 by exploring post-treatment changes in body weight and cardiometabolic risk factors following a longer (68-week) initial treatment period with semaglutide or a placebo and in the absence of active lifestyle intervention support during the 1-year off-treatment follow-up period [37]. Following treatment withdrawal, semaglutide and placebo participants regained 11.6 (SD: 7.7) and 1.9 (SD: 4.8) percentage points of lost weight by week 120, resulting in net losses of 5.6% (SD: 8.9%) and 0.1% (SD: 5.8%), respectively, from week 0 to week 120. Cardiometabolic improvements were seen from week 0 to week 68 with semaglutide reverted towards the baseline at week 120 for most variables [37].

**Table 1:** Studies that specifically looked at weight regain in a randomized fashion.

Trial Information	Period and Country	Type of Study	Inclusion/Exclusion Criteria	Design	Results
Liraglutide					
Author(s): Xavier Pi-Sunyer et al. [36] Sponsor: Novo Nordisk, Denmark	June 2011–March 2013.	Randomized, placebo-controlled trial.	Inclusion criteria: patients 18 years of age or older who had stable BMI ≥ 30, or ≥27 if the patient had treated or untreated dyslipidemia or hypertension. Exclusion criteria: type 1 or 2 diabetes, use of medications that cause clinically significant weight gain or loss, previous bariatric surgery.	56-w. Randomized, placebo-controlled trial of 3.0 mg of liraglutide or placebo, injected s.c. once daily, as an adjunct to a reduced-calorie diet and increased physical activity. After 56 weeks, patients in the liraglutide group who did not have prediabetes at screening were randomly assigned in a 1:1 ratio to continue receiving liraglutide or to switch to placebo for 12 weeks. Patients in the placebo group continued to receive placebo.	After 56 weeks, patients in the liraglutide group had lost a mean (±SD) of 8.0 ± 6.7% (8.4 ± 7.3 kg) of their body weight, whereas patients in the placebo group had lost a mean of 2.6 ± 5.7% (2.8 ± 6.5 kg) of their body weight. Weight loss with liraglutide was maintained over 56 weeks and was similar regardless of prediabetes status. In Table 19 of the appendix of the study, we found that at week 68, 12 weeks of the follow-up period, a group who did not have prediabetes liraglutide/liraglutide: reduce weight 0.69 ± 2.58, group liraglutide/placebo 2.91 ± 3.01 group placebo/placebo: 0.28 ± 2.39.
Author(s): Carel W le Roux et al. [29] Sponsor: Novo Nordisk, Denmark	June 2011–March 2015.	Randomized, placebo-controlled trial.	Inclusion criteria: patients 18 years or older with stable BMI of at least 30 kg/m^2^, or at least 27 kg/m^2^ with treated or untreated dyslipidaemia, or hypertension, or both. Exclusion criteria: type 1 or type 2 diabetes, medications causing significant weight gain or loss, bariatric surgery, history of pancreatitis, major depressive or other severe psychiatric disorders.	Participants were randomly assigned, in a 2:1 ratio, to receive liraglutide 3.0 mg or placebo. Participants without prediabetes were on treatment for 56 weeks, followed by a 12-week re-randomized period; the results for this phase of the study have been reported by Xavier Pi-Sunyer et al. [36] We did not include participants without prediabetes in this trial. A total of 160 weeks plus the 12-week off-treatment follow-up period.	Liraglutide induced greater weight loss than placebo at week 160 while on treatment (–6.1% for liraglutide vs. −1.9% for placebo; estimated treatment difference −4.3%, 95% CI −4.9 to −3.7, *p* < 0.0001). Weight loss with liraglutide treatment was sustained over 3 years. After treatment cessation at week 160, some weight was regained in the liraglutide group, although the treatment difference was still significant at week 172 (–3.2%, 95% CI −4.3 to −2.2, *p* < 0.0001).
Semaglutide					
Author(s): Domenica Rubino et al. [30] Sponsor: Novo Nordisk, Denmark	June 2018–March 2020.	Randomized, double-blind, placebo-controlled trial.	Inclusion criteria: patients 18 years or older with at least one self-reported unsuccessful dietary effort to lose weight and with BMI of 30 or higher or a BMI of 27 or higher with at least one treated or untreated weight-related comorbidity (hypertension, dyslipidemia, obstructive sleep apnea, cardiovascular disease; type 2 diabetes was excluded) were enrolled. Exclusion criteria: hemoglobin A_1c_ of 6.5% (48 mmol/mol) or greater and a self-reported change in body weight of more than 5 kg within 90 days of screening.	All participants initially received open-label once-weekly subcutaneous semaglutide, 0.25 mg, increased every 4 weeks to the maintenance dose of 2.4 mg once weekly by week 16, and continued to week 20. Participants receiving semaglutide, 2.4 mg, at week 20 were randomized in a 2:1 ratio using a blocking schema (block size of 6) in a double-blind manner, to continue this treatment or switch to matching placebo for 48 weeks (weeks 20–68; randomized period), with a 7-week follow-up. All participants received a lifestyle intervention from week 0 to week 68.	During the 20-week run-in, mean body weight declined by 10.6%. Estimated mean weight change from week 20 to week 68 was −7.9% with continued semaglutide vs. +6.9% in participants switched to placebo (difference, −14.8 [95% CI, −16.0 to −13.5] percentage points; *p* < 0.001). No found data for body weight at week 75, end follow-up period.
Author(s): John p H Wilding et al. [37] Sponsor: Novo Nordisk, Denmark	September 2019–April 2020.	Randomized, double-blind, placebo-controlled trial.	Inclusion criteria: patients 18 years or older with a BMI of ≥ 30 kg/m^2^ or ≥27 kg/m^2^ with at least one weight-related co-morbidity and a history of at least one self-reported unsuccessful dietary effort to lose weight. To be eligible for the extension, participants were required to have completed treatment with semaglutide 2.4 mg or placebo at week 68. Exclusion criteria: type 1 or 2 diabetes and obesity pharmacotherapy 90 days or less before enrolment.	Randomized to 68 weeks of treatment with once weekly s.c. semaglutide 2.4 mg or placebo, plus lifestyle intervention. At week 68 (the end of the treatment period), participants were withdrawn from treatment (including lifestyle intervention) and followed for 7 weeks until week 75. The STEP 1 extension followed a subset of participants for an additional 45 weeks (a total of 52 weeks off-treatment) until the end-of-trial visit at week 120.	During the main treatment phase (from baseline [week 0] to week 68), semaglutide reduced body weight more than placebo; mean weight loss was 17.3% (SD: 9.3%) with semaglutide versus 2.0% (SD: 6.1%) with placebo. After treatment withdrawal, body weight regain was observed in both the semaglutide and placebo arms. Participants regained a mean of 11.6 percentage points (SD: 7.7) of body weight in the semaglutide arm versus 1.9 percentage points (SD: 4.8) in the placebo arm. The net mean body weight loss over the full duration of the main treatment phase and off-treatment extension phase (from week 0 to week 120) was 5.6% (SD: 8.9%) in the semaglutide arm versus 0.1% (SD: 5.8%) in the placebo arm.
Tirzepatide					
Author(s): Louis J. Aronne et al. [38] Sponsor: Eli Lilly and Company	March 2021–18 May 2023.	Phase 3 randomized double-blind, placebo-controlled trial.	Inclusion criteria: patients 18 years or older with a BMI ≥ 30 or ≥27 and at least one weight-related complication (hypertension, dyslipidemia, obstructive sleep apnea or cardiovascular disease). Exclusion criteria: diabetes, prior surgical treatment for obesity, treatment with a medication that reduces weight loss.	After 36 w. of open-label of tirzepatide experienced a mean weight reduction of 20.9%. At week 36, those switched to placebo experienced a 14% weight regain and those continuing tirzepatide experienced an additional 5.5% weight reduction during the 52-week double-blind period. This was followed by a 36-week follow-up period.	For the treatment regimen estimand, the mean percent change in weight from week 36 to week 88 was −5.5% with tirzepatide vs. 14.0% with placebo (difference, −19.4% [95% CI, −21.2% to −17.7%]; *p* < 0.001.

Two other studies are very closely related: the SCALE studies [29,36]. In the first, the SCALE Obesity and Prediabetes study of Xavier Pi-Sunyer et al., it was found that at week 68, 12 weeks into the follow-up period, the group that did not have prediabetes and was receiving liraglutide exhibited a further weight reduction of 0.69 ± 2.58% [36]. The group that switched from liraglutide to placebo after 56 weeks of therapy recovered 2.91 ± 3.01% of body weight in 12 weeks. Patients without prediabetes continued the study for another 160 weeks, as reported in the study by the group of Carel W le Roux [29]. After treatment cessation at week 160, some weight was regained in the liraglutide group, although the treatment difference was still significant at week 172 (–3.2%, 95% CI −4.3 to −2.2, *p* < 0.0001).

The third study, SURMOUNT 4, which compared the efficacy of tirzepatide and a placebo, reported and analyzed data collected over a period of 88 weeks [38]. This comprises 36 weeks of treatment with tirzepatide, followed by a further 52 weeks of a double-blind period in which patients were administered a placebo or continued with tirzepatide. The mean percent change in weight from week 36 to week 88 was −5.5% with tirzepatide vs. +14.0% with the placebo (difference: −19.4% [95% CI, −21.2% to −17.7%]; *p*  < 0.001). It is important to note that all five studies were sponsored by the pharmaceutical company that holds the patent for the molecule in question. This type of pharmaceutical sponsorship was identified in 11 out of the 13 articles that were included in this review [Table 1, Table 2 and Table 3].

A further four out of the included thirteen studies were found to contain data on weight recovery upon cessation of the active molecule/placebo phase [Table 2], refs. [26,39,40,41].

These studies were all phase 1, 2 or 3 studies. However, the data on weight recovery were not an outcome in these studies and therefore were not reported as such. The data can be found either in the text or in the supplements, where the weight curve in the follow-up period is mentioned. It should be noted that the data were collected solely for safety reasons. The findings of the four studies demonstrated that weight recovery was observed immediately following the cessation of therapy. The duration of the therapeutic interventions ranged over a few weeks, with follow-up periods of 5, 7 or 12 weeks [26,39,40,41].

It is evident from the nine studies that have been conducted and reported that the efficacy of the therapy in maintaining weight reduction is contingent upon the duration of the treatment [26,29,30,36,37,38,39,40,41]. However, it is noteworthy that the continuation of the therapy over several months does not appear to impede weight regain upon its cessation [Table 1 and Table 2].

More data on the effect of therapy discontinuation would be desirable because we observed a large number of phase 2 and 3 trials with a safety/follow-up period but no report of body weight at the end of the trial or whether it was measured in the protocol [20,26,42,43,44]. We believe that this point should be included from a knowledge perspective; the evolution of weight after the discontinuation of active therapy should always be reported and analyzed as an increasing number of molecules in this drug class are likely to enter the market, and dose reductions and longer intervals between treatment administrations are required in order to better plan targeted studies and future proposals for discontinuation schemes.

Finally, we found four studies that analyzed how to counteract and/or limit recovery after GLP-1 RA therapy [Table 3].

**Table 2:** Phase 2 and 3 studies with follow-up period or safety.

Trial Information	Period and Country	Type of Study	Inclusion/Exclusion Criteria	Design	Results
Liraglutide					
Author(s): Melanie J. Davies et al. [39] Sponsor: Novo Nordisk, Denmark	June 2011–January 2013.	Randomized, double-blind, placebo-controlled, parallel-group trial.	Inclusion criteria: patients ≥ 18 years, BMI ≥ 27.0 with a stable body weight (<5-kg change in the last 3 months), diagnosed with type 2 diabetes treated with diet and exercise alone or in combination with 1 to 3 oral hypoglycemic agents (metformin, thiazolidinedione, sulfonylurea). Exclusion criteria: treatment with GLP-1 RA, DPP-4 inhibitors, insulin within the last 3 months; TSH > 6 mIU/L or <0.4 mIU/L, obesity induced by other endocrinologic disorders; current or history of treatment with medications that may cause significant weight gain, within 3 months prior to screening for this trial; previous surgical treatment for obesity.	Liraglutide once daily. The starting dose of the trial drug was 0.6 mg. It was escalated by increments of 0.6 mg weekly to the treatment dose. This occurred over 2 weeks for the 1.8 mg treatment dose and 4 weeks for the 3.0 mg treatment dose. Participants were encouraged to follow a diet, with a 500-kcal/d deficit based on estimated total energy expenditure and exercise program. A 12-week observational off-drug follow-up period was included to assess treatment-cessation effects (total study length: 68 weeks).	Data available only in supplementary figures. eFigure 3: effects of treatment cessation → change (%) in body weight from baseline to week 68: placebo −2.7% (−2.8% at 56 weeks); Liraglutide 1.8 mg −3.6% (−5% at 56 weeks); Liraglutide 3.0 mg −4.7% (−6.7% at 56 weeks).
Semaglutide					
Author(s): Lone B Enebo et al. [40] Sponsor: Novo Nordisk, Denmark	July 2018–Dec 2019.	Randomized, placebo-controlled, multiple-ascending dose, phase 1b trial.	Inclusion criteria: patients 18–55 years of age with a BMI of 27–0-39–9 kg/m^2^ and who were otherwise healthy. Exclusion criteria: Individuals aged 40 years or older with an estimated 10-year atherosclerotic cardiovascular disease risk of 5% or higher at screening were excluded from the trial.	The trial included six sequential overlapping cohorts, and in each cohort, eligible participants were randomly assigned (3:1) to once-weekly subcutaneous cagrilintide (0.16, 0.30, 0.60, 1.2, 2.4, or 4.5 mg) or matched placebo, in combination with once-weekly subcutaneous semaglutide 2.4 mg, without lifestyle interventions. In each cohort, the doses of cagrilintide and semaglutide were co-escalated in 4-week intervals to the desired dose over 16 weeks, participants were treated at the target dose for 4 weeks and then followed up for 5 weeks.	In the Appendix, Figure S3, page 13, the 5 weeks of follow-up with weight regain are shown for each dosage of cagrilintide with semaglutide 2.4 mg.
Liraglutide and semagludide					
Author(s): Patrick M O’Neil et al. [26] Sponsor: Novo Nordisk, Denmark	Oct 2015–Feb 2016.	Randomized, double-blind, placebo and active-controlled, multicentre, parallel-group, dose-ranging, phase 2 trial.	Inclusion criteria: patients 18 years or older without diabetes, and with a BMI of ≥30 kg/m^2^ that was not of endocrine etiology. Eligible individuals must have undergone at least one previous unsuccessful non-surgical weight loss. Exclusion criteria: diabetes mellitus; TSH > 6 mIU/L or <0.4 mIU/L; treatment with glucose-lowering agent(s) within 90 days before screening; previous surgical treatment for obesity.	The study consisted of a 1-week screening period, 52 weeks of treatment, and a post-treatment follow-up of 7 weeks.	A prespecified analysis of observed weight change from baseline at week 59 showed slightly smaller mean reductions in the active treatment groups than at week 52 due to off-treatment weight regain. Mean changes at week 59 for semaglutide escalated on the 4-weekly schedule were −4.9% (SD 6.2; 0.05 mg) to −13.5% (7.9; 0.4 mg), for the 2-weekly escalation were −12.0% (7.9; 0.3 mg) and −15.5% (9.3; 0.4 mg), for liraglutide 3.0 mg was −7.7% (6.9), and for pooled placebo was −1.8% (5.5). A post-hoc analysis of participants still on treatment at week 52 (regardless of the treatment group) who also had week 59 data showed a positive correlation between the amount of weight regained off treatment and the amount lost from baseline to week 52.
Beinaglutide					
Author(s): Kang Chen et al. [41] Sponsor: Shanghai Benemae Pharmaceutical Corporation	September 2019–October 2020.	Multicentre, randomized, double-blind, placebo-controlled study	Inclusion criteria: patients aged 18–70 years, BMI of 28 kg/m^2^ or higher or a BMI of 24–27.9 kg/m^2^ with weight-related co-morbidities Exclusion criteria: diabetes; use of weight-loss drugs; weight-reduction surgery; psychiatric disorder.	16-week beinaglutide or placebo (4-week dose escalation and 12-week dose maintenance) regimen subcutaneously, and underwent a 12-week post-treatment observation.	The mean body weight change at week 16 was −6.0% and −2.4% in the beinaglutide and placebo groups, respectively (treatment difference: −3.6%; 95% CI: −4.6% and −2.6%; *p* < 0.0001). Beinaglutide resulted in a weight regain rate of 0.78% during the post-treatment observation (12 weeks).

**Table 3:** Studies that analyzed how to counteract and/or limit recovery after GLP-1 therapy.

Trial Information	Period and Country	Type of Study	Inclusion/Exclusion Criteria	Design	Results
Liraglutide					
Author(s): Simona Ferjan et al. [45] Sponsor: Ministry of Health, Republic of Slovenia, Tertiary Care Scientific grant number 20120047 of the University Medical Centre Ljubljana.	Published in 2017.	Prospective randomized open-label study.	Inclusion criteria: type A phenotype of PCOS, including the concomitant presence of hyperandrogenemia on either the biochemical or the clinical level, menses abnormalities, and PCO morphology. Aged 18 years or older to menopause and obese (BMI ≥ 30). Exclusion criteria: significant cardiovascular, kidney or hepatic disease, personal or family history of medullary thyroid carcinoma, known history of gallbladder disease or pancreatitis.	Pretreated with liraglutide 3.0 mg for 12 weeks. After stopping liraglutide, they were switched to metformin (MET) 1000 mg twice daily (BID) alone (n = 12) or combined treatment (COMBO) with metformin 1000 mg twice daily and sitagliptin 100 mg daily (QD) (n = 12). Lifestyle intervention was reinforced after liraglutide cessation in both groups. Reducing diet of 500–800 kcal/. A total of 30 min of moderate-intensity physical activity daily was promoted. The second part of the treatment (after stopping liraglutide) lasted 12 weeks.	Before randomization, the average weight loss induced with 12-week treatment with liraglutide 3 mg was 5.1–3.6 kg. Weight regain in 12 weeks after liraglutide cessation did not correlate with weight loss achieved with liraglutide before randomization. Subjects treated with MET alone regain on average 4.7 ± 2.7 kg (*p* = 0.002) compared with 0.9 ± 2.5 kg in COMBO group (*p* = 0.147). BMI increased by 1.7 ± 0.9 kg/m^2^ in MET arm (*p* = 0.002) compared with statistically insignificant increase of 0.3 ± 0.8 kg/m^2^ in COMBO.
Author(s): Joan Khoo et al. [46] Sponsor: National Medical Research Council of Singapore	September 2014–July 2016.	Prospective randomized pilot study.	Inclusion criteria: Asians patients non-diabetic, abdominally obese (BMI > 28 kg/m^2^, waist circumference [WC] ≥ 90 cm in men or ≥80 cm in women), diagnosed with NAFLD and steatohepatitis, in the absence of other causes of hepatic steatosis and chronic liver disease. Exclusion criteria: history of excessive alcohol intake; weight-loss medications.	Structured combined diet-exercise programme (DE) group vs. Liraglutide (LI) group. 26 weeks of active weight loss phase (weeks 0–26) followed by 26 weeks of weight maintenance phase (weeks 27–52).	DE and LI groups had significant (*p* < 0.01) and similar reductions in weight (−3.5 ± 3.3 vs. −3.0 ± 2.2 kg), LFF (−8.1 ± 13.2 vs. −7.0 ± 7.1%), serum alanine aminotransferase (−39 ± 35 vs. −26 ± 33 U/L) and caspase-cleaved cytokeratin-18 (cCK-18) (−206 ± 252 vs. −130 ± 158 U/L) at 26 weeks. At 52 weeks, the LI group significantly (*p* < 0.05) regained weight (1.8 ± 2.1 kg), LFF (4.0 ± 5.3%) and cCK-18 (72 ± 126 U/L), whereas these were unchanged in the DE group.
Author(s): Camilla K. Svensson et al. [47] Sponsor: Supported by an unrestricted grant (Dr. Fink-Jensen) and Novo Nordisk A/S, Capital Region Psychiatry Research Group, The foundation of King Christian X, and grants from the Lundbeck Foundation (Dr. Jespersen and Dr. Svensson).	May 2013–February 2016.	Randomized, double-blinded, placebo-controlled trial.	Inclusion criteria: overweight/obese patients with prediabetes, diagnosed with a schizophrenia-spectrum disorder and treated with clozapine or olanzapine. Exclusion criteria: type 1 and type 2 diabetes, other serious somatic illnesses.	Randomly assigned to receive either liraglutide or placebo At baseline and every four weeks until the end of treatment (week 16), and at the one-year follow-up (week 68). Changes in medication, blood tests, psychiatric and somatic diagnoses, and diet and exercise habits were recorded. Treatment between 16 and 68 weeks was by clinician’s choice.	One year after the end of treatment (68 weeks from baseline) the liraglutide group (within-group analyses) had a significant increase in body weight, BMI, waist circumference, LDL and HDL. Compared to the placebo group (between-group analyses), the liraglutide group maintained a significant body weight loss of 3.8 kg (*p* = 0.04) and a reduction in BMI of 1.6 kg/m^2^ (*p* = 0.02) from baseline to one-year follow-up. Many drops or exclusion of patients for various reasons.
Semaglutide					
Author(s): John Blundell et al. [48] Sponsor: Novo Nordisk, Danmark	Published in 2017.	Single-center, randomized, double-blind, placebo-controlled, two-period crossover trial.	Inclusion criteria: patients 18 years or older, BMI of 30 to 45 kg/m^2^ and stable body weight. Exclusion criteria: diabetes; previous surgical treatment for obesity.	Two 12-week crossover treatment periods, separated by a wash-out period of 5 to 7 weeks. Randomized 1:1 to one of two treatment sequences: semaglutide– placebo or placebo–semaglutide.	After 12 weeks of treatment with semaglutide, a change from baseline in mean body weight of −5.0 kg was observed, vs. +1.0 kg with placebo.

The study by Simona Ferjan’s group analyzed two combinations to prevent weight regain after the initial weight loss induced by liraglutide therapy: metformin (MET) alone and the combination of metformin and sitagliptin (COMBO) [45]. A lifestyle-altering intervention was included. After liraglutide 3.0 mg cessation, no further weight loss was observed in either group. After 12 weeks, there was weight regain in both groups: the MET group regained 4.5% ± 2.5% of body weight as opposed to 0.8% ± 2.6% in the COMBO group. The study carried out by the group of Joan Khoo using liraglutide in comparison with a physical activity and dietary modification (DE group) showed that the use of pharmacological therapy alone in overweight/obese non-diabetic patients with non-alcoholic steatohepatitis (NASH) did not produce long-term results in this case [46]. This is because once the GLP-1 RA was stopped, half the weight was regained over a short period (−3.03 ± 2.43 kg in 26 weeks), as was also observed in the DE group. However, the liraglutide group recovered 1.78 ± 2.17% in 26 weeks, which did not occur in the DE group (−4.01 ± 3.80 but recovered to −0.36 ± 3.32; *p* = 0.99). The difference in the initial weight loss was not significant between the groups, but the weight regain was (*p* < 0.05). The latter data confirm what was already known: pharmacological therapy alone with a GLP-1 RA or a similar molecule, without associated physical activity or dietary modification, does not lead to the maximum weight loss potential of the molecule [22].

## 4. Discussion

The aim of this review is to analyze the effect of GLP-1 RA therapy compared to placebo on the post-drug weight trajectory, in order to better understand its potential and role in the treatment process of obese patients.

This new therapeutic option, which has recently entered the market, has rapidly gained significant interest and awareness, largely due to its widespread use on social media platforms [49]. The use of GLP-1 RAs has experienced a remarkable surge worldwide, largely due to their perceived efficacy, minimal invasiveness, widespread acceptance and lack of significant objections, but the problem of weight regain after cessation of therapy with GLP-1 RAs, as demonstrated in some individual studies, has been identified in clinical practice. Therefore, a comprehensive analysis and study of this phenomenon was considered essential to mitigate or prevent it.

Due to its complexity, obesity is now recognized as a multifactorial chronic disease: it can derive from a combination of both environmental, behavioral, genetic and hormonal factors [50]. It is well-documented that obesity provokes significant hormonal changes; these also undergo alterations during the process of weight reduction [51,52]. Reducing body weight is a beneficial outcome because it treats and improves chronic weight-related diseases, including diabetes, as well as cardiovascular risk, arthritic pain and the risk of developing certain types of cancer [53,54,55,56]. Recent studies have shown a rebound effect when weight loss is stopped, as this could be caused by a physiological homeostatic process that leads to the regain of the lost weight [15,52]. The issue of weight regain in obese patients is a well-documented problem.

Obesity is the result of multiple factors, and consequently, weight regain is influenced by different factors in each individual patient [15]. Therefore, it is difficult to believe that a single molecule, despite having pleiotropic and diverse effects in different parts of the body (such as GLP-1 RA, which has receptors both in the gastrointestinal tract and in the central nervous system), can solve such a complex disease and, above all, triumph definitively where other molecules have failed, i.e., in preventing weight regain when their use is stopped [57,58]. The challenge of preventing or managing post-treatment weight gain, which varies in intensity depending on the therapeutic intervention administered, remains unresolved. With the current state of the art, it is also difficult to obtain homogeneous literature data on the exact incidence of this phenomenon, and even more difficult to identify possible therapeutic schemes to ensure that it does not occur.

Guidelines for the clinical management of people with obesity have generally lagged behind the rapid development of newer pharmacological therapies. Although lifestyle modification, such as diet improvement and physical activity, still constitutes the cornerstone of obesity management, its ineffectiveness when implemented in isolation has been well documented [7,8,15]. The efficacy of the intervention is defined as a minimum one-year weight loss of 2–5%. The contemporary body of evidence underscores the necessity for a multidisciplinary approach to the management of obesity, whereby a dedicated team of healthcare professionals collaborates with the patient over an extended period, utilizing a range of therapeutic interventions, including metabolic surgery and pharmacological drugs, which are currently recognized and approved by most global authorities for the treatment of obesity: orlistat, naltrexone-bupropion, phentermine-topiramate, the most recent ones tirzepatide, liraglutide and semaglutide [7,15]. With the classic drugs, however, weight regain is well documented: orlistat and lorcaserin have shown efficacy, albeit limited, in weight loss, but the data show that they do not prevent weight gain after cessation [13,14].

Metabolic surgery has been demonstrated to be cost-effective as well as efficacious in the treatment of obesity and in the management of certain co-morbidities, including the possibility of inducing remission of type 2 diabetes [9,59]. Nevertheless, it is not readily accepted by obese patients, as it is an invasive procedure [10]. Its efficacy in promoting weight loss, even in the short term, appears to surpass that of all other molecules, including those that have been found to be the most efficacious, such as Tirzepatide: studies have shown that mean total weight loss is approximately 30% after one year of bariatric surgery, 31% for Roux-en-Y gastric bypass (RYGB), 25% for sleeve gastrectomy, compared to only a mere 6% to 20% of total weight loss in patients undergoing an annual treatment with GLP1-RA [29,38,60,61]. Furthermore, data has been collected that demonstrates the effectiveness of the treatment over time: in a systematic review and meta-analysis of studies with 10 or more years of follow-up, 18 reports of RYGB showed a weighted mean of 56.7% of excess body weight (EWL), 2 reports of SG showed 58.3% EWL [62]. However, even this therapeutic option is not immune to the issue of weight regain. The quantification of weight regain post-bariatric surgery is not uniformly defined. Consequently, reliable estimation is challenging. Typically, this process commences in the second postoperative year. Athanasiadis et al. state that in one out of six cases, patients regain more than 10% of the weight lost [63]. Once more, there is an absence of both clear and consistent guidelines for management and treatment. For this reason, it is essential that each patient is assessed on an individual basis. One of the potential options that is currently being studied involves the subsequent introduction of therapy with GLP-1 RA [64]. In such cases, novel research questions emerge, including the optimal duration of therapy and, most importantly, the potential consequences of its interruption. Will there be a new weight gain? Indeed, we are confronted with a novel scenario: patients who have regained weight following metabolic surgery, recognized as the most efficacious therapeutic option in the management of obese patients. The present situation is not congruent with that wherein patients who had undergone surgery or pre-treatment with a GLP-1 RA were systematically excluded from participation.

With regard to GLP-1 RAs, current data suggest that they are more effective in terms of weight loss than those currently on the market [29,30,36,37,38,39]. However, a potential deterrent to their use may be the fact that at present they are only available in injectable form. It is evident that numerous pharmaceutical manufacturers are prepared to introduce novel oral medications into the market [24]. The weight-reducing efficacy of these classes of medications is evident (6–20% at 52 weeks); however, all studies reviewed revealed that weight regain occurred following cessation of treatment [29,30,36,37,38]. The paucity of data regarding weight regain has been noted in the literature, which has been described as being scattered and poorly defined. However, the present review has sought to address this gap by providing a clear and comprehensive overview of the subject. The five most significant randomized studies (see Table 1) demonstrate that weight regain occurs following the cessation of therapy, irrespective of the duration of treatment, whether it is brief or protracted. However, it should be noted that the collection of these data is not always reported, and in some cases, they are not easily identifiable. For instance, in certain studies, these data can only be found by examining the tables in the appendices [40]. The efficacy of the new therapy is evident in the significant weight loss observed, which exceeds the range of 10–20% compared to the 5–10% range typically achieved with conventional methods.

In the STEP 1 study, mean weight loss at 68 weeks was 17.3% with semaglutide versus 2.0% with placebo. Participants regained an average of 11.6% points of body weight in the semaglutide group versus 1.9% points in the placebo group [37]. The above points can be summarized as follows: the mean net weight loss over the entire duration of the main treatment phase and the off-treatment extension phase (from week 0 to week 120) was 5.6% in the semaglutide group versus 0.1% in the placebo group. In STEP 4, always with semaglutide, mean body weight decreased by 10.6% during the 20-week run-in period [30]. The estimated mean weight change from week 20 to week 68 was −7.9% with semaglutide continuation versus +6.9% in participants who crossed over to placebo. No data were found for body weight at week 75, the end of the follow-up period [30]. In the SCALE Obesity and Prediabetes, liraglutide induced greater weight loss than placebo at week 160 while on treatment: −6.1% for liraglutide versus −1.9% for placebo [29]. Weight loss with liraglutide was maintained for 3 years. After stopping treatment at week 160, some weight was regained in the liraglutide group, although the treatment difference was still significant at week 172 (−3.2%) [29]. This finding supports the idea that obesity is a complex and chronic disease and that short-term interventions with these drugs are unlikely to change the course of the disease [15].

Thus, the weight-loss efficacy of GLP-1RAs has been demonstrated in both randomized, placebo-controlled trials and real-world studies [65]. The results of the latter group are more relevant to clinical practice, but these studies show a lower efficacy in terms of weight loss. It is clear that there is considerable variability in the efficacy of GLP-1 RAs therapy. Indeed, not all molecules are equally effective in inducing weight loss in a significant proportion of patients. For example, liraglutide shows clear inferiority to both semaglutide and tirzepatide [23,36,61]. Furthermore, of particular importance in assessing the efficacy is the dose of the molecule, as evidenced by the results of phase 2 and phase 3 trials, which show that the efficacy of different molecules in promoting weight loss is predominantly influenced by the dose administered [27,28].

The ultimate benefit of absolute weight loss compared with placebo is seen within a transient period, albeit at levels that are likely to have a minimal impact on prognosis [21]. The study by Pi-Sunyer’s group was continued for 12 weeks after the initial 56 weeks of liraglutide therapy to re-evaluate patients without prediabetes and to determine the effects of stopping treatment [36]. The results showed an increase in both weight and fasting plasma glucose. In the SURMOUNT-4 study, a significant reduction in body weight (9.9%) was observed in patients who were switched to a placebo for 1 year after 36 weeks of tirzepatide treatment [38]. However, a significant reversal of the initial improvement in cardiometabolic risk factors was observed in the group that discontinued therapy after 88 weeks, with less pronounced weight loss (25.3% overall) and loss of the previously observed health benefits. The maximum tolerated dose of tirzepatide was administered during the study, and the safety profile observed was consistent with that previously reported in the SURMOUNT and SURPASS trials, as well as studies of incretin-based therapies approved for the treatment of obesity and overweight [38,66]. Although adverse effects of GLP-1RAs have been reported with high frequency in both randomized and real-world studies, these effects are primarily gastrointestinal in nature, including nausea, constipation and diarrhea. The initial hypothesis that pancreatitis or pancreatic cancer might be of concern appears to have been refuted by meta-analyses and numerous large real-world observational studies [67,68].

This review draws the attention of the scientific community to the fact that obesity is a chronic disease for which therapeutic tools exist, such as bariatric surgery and various pharmacological therapies that are effective in reducing weight. However, these treatments have an intrinsic problem that underlies their use, namely weight regain once the effect wears off (whether due to cessation of drug administration or to re-expansion of the gastric pouch remaining after bariatric surgery). It is important to recognize the significant economic pressures on all healthcare systems worldwide. Consequently, cost–benefit analyses of different therapeutic approaches should be carried out systematically. However, conducting such studies is challenging due to the variability in healthcare costs and drug therapies across countries. Anyway, this author believes that they should always be carried out, especially in the context of new therapies that appear promising but for which long-term data are not yet available, as in the case of GLP-1 RAs. This is for ethical reasons, particularly in countries where public healthcare covers the cost of such treatments. A notable aspect of this analysis relates to the need to conduct a comparative study with surgery, as current evidence suggests that the former approach has superior long-term weight loss maintenance outcomes compared to non-surgical methods. A single study has suggested that endoscopic sleeve gastroplasty may be a more cost-effective alternative to medical therapy compared with semaglutide [69].

In conclusion, it is imperative that the criteria for determining which patients demonstrate a superior response to therapy be more precisely defined. Indeed, as shown by the results of numerous studies, there is a subgroup of patients who have no response, a moderate response, or a favorable response [27,28,70]. In addition, it is important to determine whether there are categories of patients who are more susceptible to significant weight regain after discontinuation of therapy. The goal remains to prevent weight regain.

It is imperative that all the points highlighted by the review are clarified and further studies are needed to understand the potential long-term benefits and risks (i.e., residual effects) of such long-term and short-term therapy. Further research is needed to determine the most effective way to stop therapy, which would open up a new research landscape for new trials. However, it is unclear whether it would be better to gradually reduce the dose, increase the interval between doses, or develop new drugs. Another possibility is the combination of additional drugs or their sequential use.

## 5. Conclusions

The analysis showed that there is a rapid regain of weight after cessation of therapy, whether short-term or long-term, with a GLP-1 RA or a GIP/GLP-1 RAs. This rebound is likely to significantly reduce the metabolic benefits of weight loss. Given the efficacy of these molecules, it is essential that future research focuses on elucidating the optimal duration of these treatments or identifying techniques or regimens that involve dose reduction to prevent weight regain.

## Data Availability

Not applicable.

## Abbreviations

The following abbreviations are used in this manuscript:

HRQoL	Health-related quality of life
QALYs	Quality-adjusted life-years
BMI	Body mass index
EOC	Ente Ospedaliero Cantonale
PRISMA	Preferred Reporting Items for Systematic Reviews and Meta-analyses
s/c	subcutaneously
GLP-1 RA	Glucacon-like peptide 1 receptor agonists
COPD	Chronic obstructive pulmonary disease
RYGB	Roux-en-Y gastric bypass
SG EWL	Sleeve gastrectomy Excess body weight loss
GIP	Gastric inhibitory polypeptide
PCOS	Polycystic ovary syndrome
